# Identification of the *Hevea brasiliensis* AP2/ERF superfamily by RNA sequencing

**DOI:** 10.1186/1471-2164-14-30

**Published:** 2013-01-16

**Authors:** Cuifang Duan, Xavier Argout, Virginie Gébelin, Marilyne Summo, Jean-François Dufayard, Julie Leclercq, Kuswanhadi, Piyanuch Piyatrakul, Julien Pirrello, Maryannick Rio, Antony Champion, Pascal Montoro

**Affiliations:** 1 CIRAD, UMR AGAP, Montpellier, F, 34398, France; 2 CATAS, RRI, Hainan, Danzhou, 571737, China; 3 IRRI, Sembawa Research Centre, P.O. Box 1127, Palembang, Indonesia; 4 Rubber Research Institute, Chatuchak, Bangkok, 10900, Thailand; 5 IRD, UMR DIADE, Laboratoire LCM, Dakar, Senegal

**Keywords:** Ethylene, Rubber, Transcription factor, Transcriptome

## Abstract

**Background:**

Rubber tree (*Hevea brasiliensis*) laticifers are the source of natural rubber. Rubber production depends on endogenous and exogenous ethylene (ethephon). AP2/ERF transcription factors, and especially Ethylene-Response Factors, play a crucial role in plant development and response to biotic and abiotic stresses. This study set out to sequence transcript expressed in various tissues using next-generation sequencing and to identify AP2/ERF superfamily in the rubber tree.

**Results:**

The 454 sequencing technique was used to produce five tissue-type transcript libraries (leaf, bark, latex, embryogenic tissues and root). Reads from all libraries were pooled and reassembled to improve mRNA lengths and produce a global library. One hundred and seventy-three AP2/ERF contigs were identified by *in silico* analysis based on the amino acid sequence of the conserved AP2 domain from the global library. The 142 contigs with the full AP2 domain were classified into three main families (20 AP2 members, 115 ERF members divided into 11 groups, and 4 RAV members) and 3 soloist members. Fifty-nine AP2/ERF transcripts were found in latex. Alongside the microRNA172 already described in plants, eleven additional microRNAs were predicted to inhibit *Hevea* AP2/ERF transcripts.

**Conclusions:**

*Hevea* has a similar number of *AP2/ERF* genes to that of other dicot species. We adapted the alignment and classification methods to data from next-generation sequencing techniques to provide reliable information. We observed several specific features for the ERF family. Three HbSoloist members form a group in *Hevea*. Several *AP2/ERF* genes highly expressed in latex suggest they have a specific function in *Hevea*. The analysis of AP2/ERF transcripts in *Hevea* presented here provides the basis for studying the molecular regulation of latex production in response to abiotic stresses and latex cell differentiation.

## Background

Natural rubber accounts for 42.3% of the 23.9 million tons of rubber consumed worldwide [1]. *Hevea brasiliensis* is the sole commercial source of natural rubber. The increasing demand for natural rubber calls for improved productivity in rubber plantations. Cis-1,4-polyisoprene chains are synthesized in the rubber particles of latex cells. Rubber particles account for up to 90% of the dry matter in latex cytoplasm, which is harvested by tapping the soft bark of rubber trees [2]. Latex production depends on genetic, environmental and harvesting components. Harvesting systems use ethephon, an ethylene (ET) releaser applied to the tapping panel, to stimulate latex production by improving the flow and regeneration of latex. Tapping and ethephon stimulation frequencies are adjusted to *Hevea* clones according to their metabolism [3]. Given the high pressure in the phloem tissue, latex is expelled after tapping. Tapping and ethephon are likely to be sources of stress conducive to the production of secondary metabolites and consequent rubber, but over a certain stress limit they lead to tapping panel dryness (TPD) [4]. Mechanical wounding and osmotic stresses related to tapping trigger the production of endogenous ethylene and oxylipins such as jasmonic acid (JA) [5,6]. Both mechanical wounding and methyl-jasmonate treatments induce the differentiation of secondary latex cells in extended young stems [7-9]. In trees, secondary latex cells are differentiated from cambium and then anastomosed to create laticifer vessels [10]. Ethephon application also induces several biochemical processes in laticifers, such as sucrose loading, water uptake, nitrogen assimilation or synthesis of defence proteins [11,12], involving a large number of ethylene-response genes [13-18], whereas its direct role in rubber biosynthesis is controversial [19].

Given the major role of ethylene and jasmonic acid in regulating latex cells, Ethylene-Response Factors are greatly expected to be involved in latex cell functioning. Indeed, ET and JA signalling pathways involve transcription factors such as Ethylene-Responsive Element Binding Proteins (EREBP), also called the Ethylene-Response Factors (ERF) family [20]. ERFs have been shown to act as activators or repressors of additional downstream ethylene responsive genes. ERFs are a control point for crosstalk with other signals and they function as an integrator of the ethylene and jasmonic acid pathway [21]. Multiple signalling pathways converge on ERFs by transcriptional and post-transcriptional regulation [22]. Ethylene and jasmonate pathways converge in the transcriptional activation of ETHYLENE RESPONSE FACTOR1 (ERF1), which regulates *in vivo* the expression of a large number of genes responsive to both ethylene and jasmonate. ERF1 acts downstream of the intersection between the ethylene and jasmonate pathways suggesting that this transcription factor is a key element in the integration of both signals for the regulation of defence response genes [23,24]. In biotic stress, AP2/ERF transcription factor ORA59 acts as the integrator of the JA and ET signalling pathways and is the key regulator of JA- and ET-responsive PDF1.2 expression [21,25].

The ERF family was first discovered in *Nicotiana tabaccum* by Ohme-Takagi and Shinshi [20]. The ERF family is one of the largest families of transcription factors with 122 genes out of the 2016 predicted transcription factors from 58 families in *Arabidopsis*[26,27]. The ERF family belongs to the AP2/ERF superfamily. This superfamily encodes transcriptional regulators that serve a variety of functions in plant development and responses to biotic and abiotic stimuli [28-30]. Members of the AP2/ERF superfamily contain at least one AP2 domain, which consists of about 60 amino acids. This domain is involved in DNA binding to a conserved AGCCGCC sequence called the GCC-box [20,31] or to a dehydration response element (DRE: TACCGACAT) containing the C-repeat [32,33]. The structure of the AP2 domain was first reviewed by Riechmann and coll. [34]. Initially, the *APETALA2* (*AP2*) gene was isolated by T-DNA insertional mutagenesis in *Arabidopsis*[35]. This gene encodes a 432-amino acid protein with two copies of a 68-amino acid direct repeat called the AP2 domain. The AP2 domain consists of three anti-parallel β-sheets and one α-helix. Two conserved elements, YRG and RAYD, have been identified. The latter is an 18-amino acid core region that is predicted to form an amphipathic α-helix [36]. In some AP2 genes, the AP2 domain contains a 37-amino acid serine-rich acidic domain putatively functioning as an activation domain, and a 10-amino acid domain including a putative nuclear localization sequence KKSR [35]. While previously thought to be plant-specific transcription factors, AP2 domain-containing genes were recently found in bacteria and viruses, which are predicted to be HNH endonucleases [37,38].

Several ways of classifying the AP2/ERF superfamily have been proposed in plants. Although all of them are based on the number of AP2 domains, some differences exist. Firstly, Sakuma *et al.* described five subfamilies including AP2, RAV, Dehydration Responsive Element Binding Proteins (DREB), Ethylene-Responsive Element Binding Proteins (EREBP), also called the Ethylene-Response Factors (ERF) family [20], and others based on a homology of the DNA binding domain, and the DNA sequences that bind it, namely the DRE element or GCC-box separately [39]. The AP2, ERF/DREB and RAV subfamilies have two AP2 domains, one AP2 domain, or one AP2 and one B3 domain, respectively. Groups A1 to A6 and B1 to B6 have been assigned to the DREB and ERF families [39]. Secondly, Nakano *et al.* classified these proteins in only three major families: AP2, ERF and RAV [27]. The ERF family was then divided into ten groups according to the structure of the AP2 domain, with groups I to IV corresponding to the DREB subfamily in Sakuma’s classification. To date, Nakano’s classification method has remained a reference for organizing the AP2/ERF superfamily in three families (AP2, ERF, RAV) and the ERF family in ten groups. In the construction of phylogenetic trees, methods for multiple sequence alignment and tree reconstruction have to be considered with caution. In the analyses by Sakuma and Nakano, ClustalW followed by a neighbor-joining method was chosen. Currently, although computationally intensive, the multiple sequence alignment software MUSCLE followed by a maximum likelihood method (PhyML) is more relevant [40-44].

The availability of the whole genome sequence of several plant species has made it possible to confirm a relatively well-conserved organization of the AP2/ERF superfamily with 147, 149, 202, 180 and 146 genes in *Arabidopsis thaliana, Vitis vinifera, Populus trichocarpa Oryza sativa,* and Solanum lycopersicon, mostly represented by the ERF family [27,28,45-47]. Transcript sequencing is also an alternative for identifying such gene families. For instance, 156 *AP2/ERF* genes consisting of 148 ERFs, 4 AP2s and 4 RAVs were identified in *Gossypium hirsutum* from EST databases [48]. In *Hevea brasiliensis*, transcriptome sequencing has been carried out on latex, bark, leaf and shoot apex tissues using various methods [49-54]. A few number of ERF sequences have been released in the Genbank (HbEREBP1: HQ171930.1; EREBP2: HQ171931.1; DREB1p: HQ902146.1; CBF1: AY960212.1) [6]. As preliminary experiment, we identified AP2/ERF unigenes from latex and leaf tissues of the *Hevea* clone Reyan 7-33-97 members [52]. This analysis revealed 45 AP2/ERF with partial AP2 domain that did not allow gene classification (Additional file 1). Given the involvement of wounding, jasmonate and ethylene in natural rubber production, we developed a reference transcriptome that covers a large number of tissues and environmental conditions to have a fully comprehensive *Hevea* transcriptome and we examined the organization of the AP2/ERF superfamily in *Hevea*. Firstly, RNAs were isolated from different tissues of plants at several stages of development growing under various conditions, and transcripts were sequenced using GS-FLX next-generation sequencing (NGS) technologies. Secondly, contigs harbouring at least one AP2 domain were identified in tissue-type libraries for leaves, bark, latex, roots and embryogenic tissues and from a global library which pooled reads from all tissue-type libraries. AP2 domain-containing genes were aligned with the *Arabidopsis* AP2/ERF sequences and classified according to Nakano’s method based on a phylogenetic analysis of the conserved AP2 domain, which was optimized using a maximum likelihood method (PhyML) [40-42]. Post-transcriptional regulation was checked by predicting microRNA-targeted *AP2/ERF* genes. This study suggested that some *HbAP2/ERF* genes expressed in latex cells could be involved in specific biological processes.

## Results

### Transcript sequence libraries

**Figure 1:**
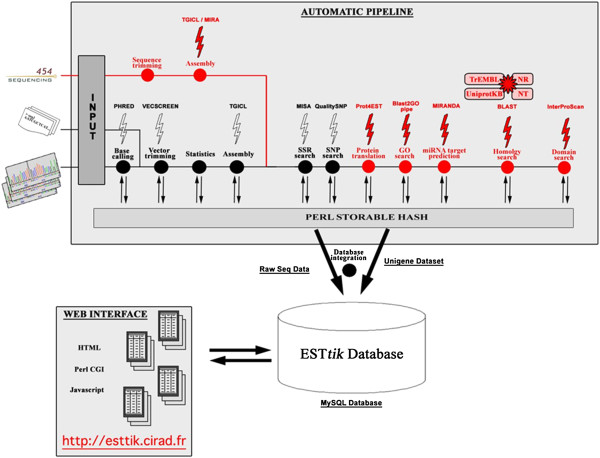
**Automated pipeline for the analysis of 454 sequence data and their assembly.** In red: new modules for the analysis of 454 data and new annotation procedure. Generated contigs for each library were stored in the ESTtik database http://esttik.cirad.fr/.

Transcript sequences were produced for five tissue-type libraries (embryogenic tissues, leaf, bark, latex and root) from the *Hevea* clone PB 260 by the pyrosequencing GS-FLX 454 technique. Total mRNAs were isolated from different tissues collected from plants at different stages of development and having undergone different treatments in order to have the most complete representation of the transcriptome (Table 1). A half run of 454 sequencing generated more than 500,000 reads for each tissue-type library (Table 2). An automatic pipeline was used to remove reads shorter than 120 bp and non-coding sequences and for clustering and assembly of contigs with TGICL (Figure 1). The annotation of contigs has been proceeded using miR target prediction by MIRANDA, and protein function by similarity with several protein sequence databases by BLAST. For the embryogenic tissues, leaf, bark, latex and root libraries, the number of contigs was 44,988, 29,910, 45,114, 29,016 and 50,146 respectively (Table 2). Reads from all libraries (2,390,118) were re-assembled in a global library to generate 94,981 contigs. The large coverage of the global library led to improve contig lengths, which reached 807 bp on average.

**Table 1:** List of plant materials and treatments used to isolate total RNA

**Plant material**	**Treatment**	**Condition**	**Collected tissues for the library**
Callus	MM medium	2 weeks	Embryogenic tissue
Embryogenic callus	EXP medium	4 weeks	
Somatic embryo	DM medium	8 weeks	
1-month-old in vitro plant	None	-	Leaf, root
1-year-old in vitro plant	None	-	Leaf, bark, root
	Wounding	1, 4 and 24 hours	
	Ethylene	1 ppm for 4, 8 and 24 hours	
	Water deficit	3 weeks	
	Flood	72 hours	
	Cold	24 hours	
4-year-old in vitro plant	None	-	Root
3-month-old shoot from budded plant	None	-	Leaf, bark
	Wounding	1, 4 and 24 hours	
	Ethylene	1 ppm for 4, 8 and 24 hours	
	Methyl-jasmonate	0.3 μM for 1, 4 and 24 hours	
5-year-old tree	Non-tapped	-	Latex, bark
	Tapped	Every 2 days	
	Tapped, ethephon	Every 2 days, 2.5% once a month	

RNA sequencing was carried out on a mix of RNAs from the same tissues of various plant materials grown under specified treatments. MM, EXP and DM media are used for callus proliferation, somatic embryogenesis induction and embryo development, respectively. All media are described in [55].

**Table 2:** Summary of 454 sequencing and clustering using automatic pipeline for various tissue-type libraries (embryogenic tissues, leaf, bark, latex, and roots) and a global library combining reads from all tissues

	**Embryogenic**	**Leaf**	**Bark**	**Latex**	**Root**	**Global**
**Sequence in input**	516,589	574,763	545,237	576,497	605,730	-
**Discarded reads**	98,364 (19%)	123,858 (22%)	55,035 (10%)	93,758 (16%)	54,396 (9%)	-
Short reads (< 120 bp)	95,771	122,639	52,275	93,246	51,761	-
Non-coding sequence	2,593	1,219	2,760	512	2,635	-
**Good quality reads**	418,225 (81%)	450,905 (78%)	490,202 (90%)	482,739 (84%)	551,334 (91%)	2,390,118
Max length (bp)	925	582	614	880	641	925
Min length (bp)	120	120	120	121	120	120
Average length (bp)	293	282	399	245	395	325
**Assembly**						
Number of contigs	44,988	29,910	45,114	29,016	50,146	94,981
Maximum length of contigs (bp)	3,461	2,062	4,280	2,302	4,134	6,657
Minimum length of contigs (bp)	83	113	97	121	93	62
Average length of contigs (bp)	519	552	753	423	754	807

### Identification and classification of AP2/ERF superfamily genes in *hevea*

**Figure 2:**
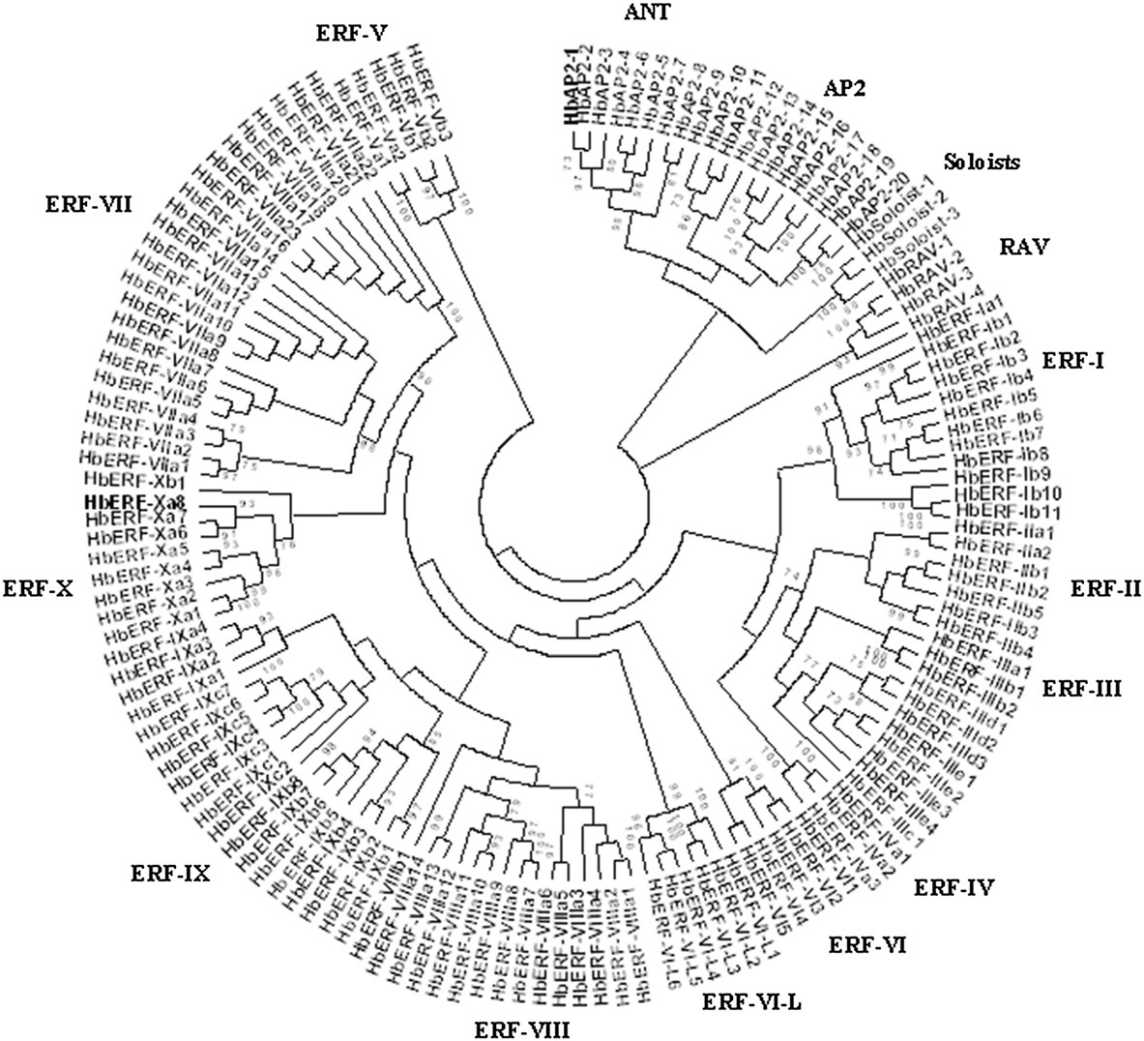
**Phylogenetic tree of *Hevea *AP2/ERF proteins.** The amino acid sequences of the AP2 domain were aligned using Muscle (Additional file 1), and the phylogenetic tree was constructed using the PhyML with an LG + T model. The families and groups to which the 142 AP2/ERF proteins belong are shown.

*Hevea* AP2/ERF transcripts were identified using TBLASTN to search for translated nucleotide in the global *Hevea* transcript library using the amino acid *Arabidopsis thaliana* AP2 domain sequences as the query. The 173 putative AP2/ERF superfamily contigs identified in the global transcript sequence database contained full-length and partial transcripts. Of them, 142 contigs had the full-length AP2 domain sequences of 58–59 amino acids. Multiple alignment analysis was performed on full-length AP2 domain sequences from *Hevea, Arabidopsis* and *Populus*. Group classification was firstly achieved by constructing the general phylogenetic tree of AP2 domains in *Arabidopsis* and *Hevea* with the neighbour-joining method (data not shown), and then the phylogenetic relationships between these genes were analysed by constructing another phylogenetic tree using the PhyML method only for *Hevea* (Figure 2). The Nakano classification method was used to organize the *Hevea* AP2/ERF superfamily into families and groups. The alignments indicated three clusters corresponding to the AP2, ERF and RAV families, with the ERF family divided into eleven major groups including an additional VI-L group, and the three soloists rooted with the AP2 family. The AP2 family was organized in two groups including eight AINTEGUMENTA (*ANT*) and twelve *AP2* genes.

The number of members of the *Hevea* AP2/ERF superfamily was compared with six other species (Table 3). The AP2/ERF superfamily has a similar number of genes in *Vitis* (149) and *Arabidopsis* (147). This number is higher for *Gossypium* (156), *Populus* (202) and *Hevea* (173), while it is lower for *Solanum* (112) and *Triticum* (117). These differences were mostly induced by a change in the number of ERF genes. In *Hevea*, twenty-five genes were assigned to the AP2 family based on the identity of their amino acid sequences with the *A. thaliana* AP2 proteins and the presence of a double AP2 domain in their sequences. This number included contigs with one or two partial AP2 domains. Ten genes containing a single complete/partial AP2 domain were classified in the AP2 family given their greater homology with the AP2 family. The largest family was the ERF family with 141 genes harbouring a single AP2 domain, including twenty-six contigs with a partial sequence of the AP2 domain. Four genes were classified in the RAV family, which had one single AP2 domain and one B3 domain. Three soloist genes were identified in *Hevea* whereas only one has been reported for *Arabidopsis* and *Populus*, and no soloists have been identified in *Solanum* and *Gossypium*.

**Table 3:** **Summary of the classification of the *Hevea brasiliensis *AP2/ERF superfamily compared with several species: *Arabidopsis thaliana***[27]***, Populus trichocarpa ***[28]***, Vitis vinifera***[45]**, *Solanum lycopersicum ***[56]***, Gossypium hirsutum***[48]***, Triticum aestivum***[26]

**Family**	**Conserved domain**		**Number of members in each AP2/ERF gene family from different species**						
			**Genome**			**Transcriptome**			
			***Arabidopsis***	***Populus***	***Vitis***	***Solanum***	***Gossypium***	***Triticum***	***Hevea***
AP2	Double AP2/ERF domain	Total	18	26	20	16	4	9	25
		Two full-length domains	14	26	20	11	4	9	9
		One full-length domain plus one partial domain	-	-	-	-	-	-	4
		Two partial domains						-	2
	One AP2/ERF domain	One full-length domain	4	-	-	5	-	-	7
		One partial domain	-	-	-	-	-	-	3
ERF	Single AP2/ERF domain	Total	122	169	122	93	148	104	141
		Full-length domain	122	169	122	85	148	104	115
		Partial domain	-	-	-	8	-	-	26
RAV	Single AP2/ERF domain plus one B3 domain		6	6	6	3	4	3	4
SOLOIST	Short single AP2/ERF domain		1	1	1	-	-	1	3
TOTAL NUMBER			147	202	149	112	156	117	173

These *AP2/ERF* sequences were obtained either after genome sequencing or transcriptome sequencing.

One hundred and fifteen *Hevea* genes with a full AP2 domain from the ERF family were organized in eleven groups according to the Nakano classification, including the VI-Like group (Table 4). The number of genes (115) for the *Hevea* ERF family was comparable to that for *Arabidopsis, Gossypium* and *Vitis* (122, 148 and 135, respectively). The *Hevea* ERF groups showed several characteristics. Firstly, several ERF groups and subgroups found in *Arabidopsis*, such as subgroup IIc and groups IVb, Xc and Xb-L, were not found in *Hevea* (Figure 2). Secondly, *Gossypium* (22 genes) and *Hevea* (23 genes) have the largest number of ERF genes for group VII and conversely they have the smallest number of genes for group IV with 2 and 3 genes, respectively for these two species.

**Table 4:** **Classification of the *Hevea brasiliensis *ERF family based on the phylogenetic analysis compared with the *Arabidopsis thaliana, Gossypium hirsutum, Populus trichocarpa *and *Vitis vinifera *species according to Nakano’s method**

	**Number of members in each group of the ERF family for different species**				
**Group**	***Arabidopsis***	***Gossypium***	***Populus***	***Vitis***	***Hevea***
I	10	9	5	5	12
II	15	17	20	8	7
III	23	25	35	22	11
IV	9	2	6	5	3
V	5	3	10	11	5
VI	8	11	11	5	5
VII	5	22	6	3	23
VIII	15	26	17	11	15
IX	17	26	42	40	19
X	8	7	9	10	9
VI-L	4	0	4	2	6
Xb-L	3	0	4	0	0
Total	122	148	169	122	115

**Figure 3:**
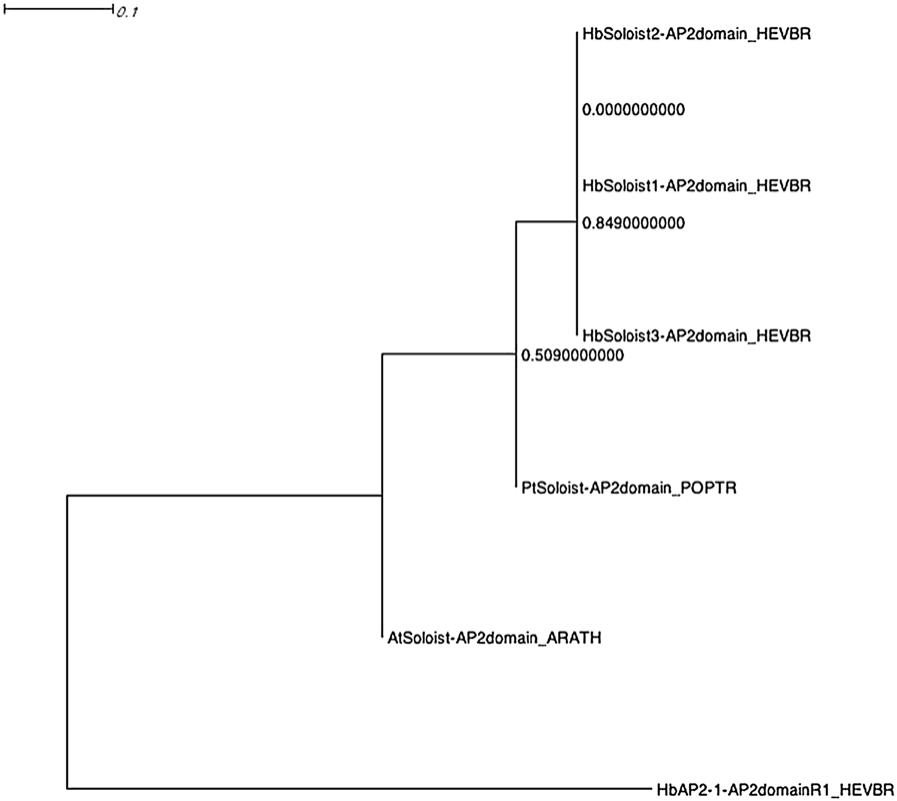
**Phylogenetic tree of *Hevea *Soloist proteins.** The amino acid sequences of the AP2 domain were aligned using Muscle, and the phylogenetic tree was constructed using the PhyML with an LG + T model.

The alignment of nucleotide and deduced amino acid sequences of the three *Hevea* soloists revealed that they shared 64.8 to 72.9% and 73.2 to 93.2% of identity, respectively (Table 5). For the AP2 domain, this identity reached 92.3 to 97.4%. A multiple alignment analysis was carried out on AP2 domain sequences from *Hevea, Arabidopsis* and *Populus* (Figure 3). This phylogenetic analysis revealed an evolution of the three *Hevea* soloists after speciation phenomena with *Arabidopsis* and more recently with *Populus*. Nakano’s classification method was compared with Sakuma’s for the 142 *Hevea* genes with a complete AP2 domain (Table 6). Families and groups were noted as subfamilies and subgroups previously by Sakuma. ERF genes were classified in two subfamilies consisting of thirty-three DREB (ERF family group I to IV) and eighty-two ERF (ERF family group V to X) genes. ERF subfamily genes were twice as large as the DREB subfamily in *Hevea*.

**Table 5:** **Identity of the three *Hevea brasiliensis *Soloists for nucleotide and amino acid residues**

**Contig**		**HbSoloist1**			**HbSoloist2**			**HbSoloist3**		
**Name**	**Length (pb)**	**Nt (%)**	**AA (%)**	**AP2 (%)**	**Nt (%)**	**AA (%)**	**AP2 (%)**	**Nt (%)**	**AA (%)**	**AP2 (%)**
HbSoloist1	1520	100	100	100	72.9	93.2	96.4	64.8	73.2	97.4
HbSoloist2	1290	72.9	93.2	96.4	100	100	100	72.6	76.8	92.3
HbSoloist3	954	64.8	73.2	97.4	72.6	76.8	92.3	100	100	100

Alignments were carried out on sequences which overlap from the full nucleotide sequences (Nt), the full deduced amino acid sequences (AA); and the amino acid sequences of AP2 domains (AP2).

**Table 6:** **Correspondence between Nakano’s and Sakuma’s classification methods for the *Hevea *AP2/ERF superfamily genes**

**Classification of the *Hevea *AP2/ERF superfamily**				
**Nakano’s method**		**Sakuma’s method**		
**Family**	**Group**	**Subfamily**	**Subgroup**	**Number of genes**
AP2 family	-	AP2	-	20
ERF	I to IV	DREB	A-1 to A-6	33
	V to X	ERF	B-1 to B-6	76
	VI-L & Xb-L		B-6	6
RAV	-	RAV	-	4
SOLOIST	-	SOLOIST	-	3
Total				142

In this presentation, AP2/ERF genes with at least one full-length domain were kept.

### Structure and group-specific residues of the AP2 domains of *ERF* genes

**Figure 4:**
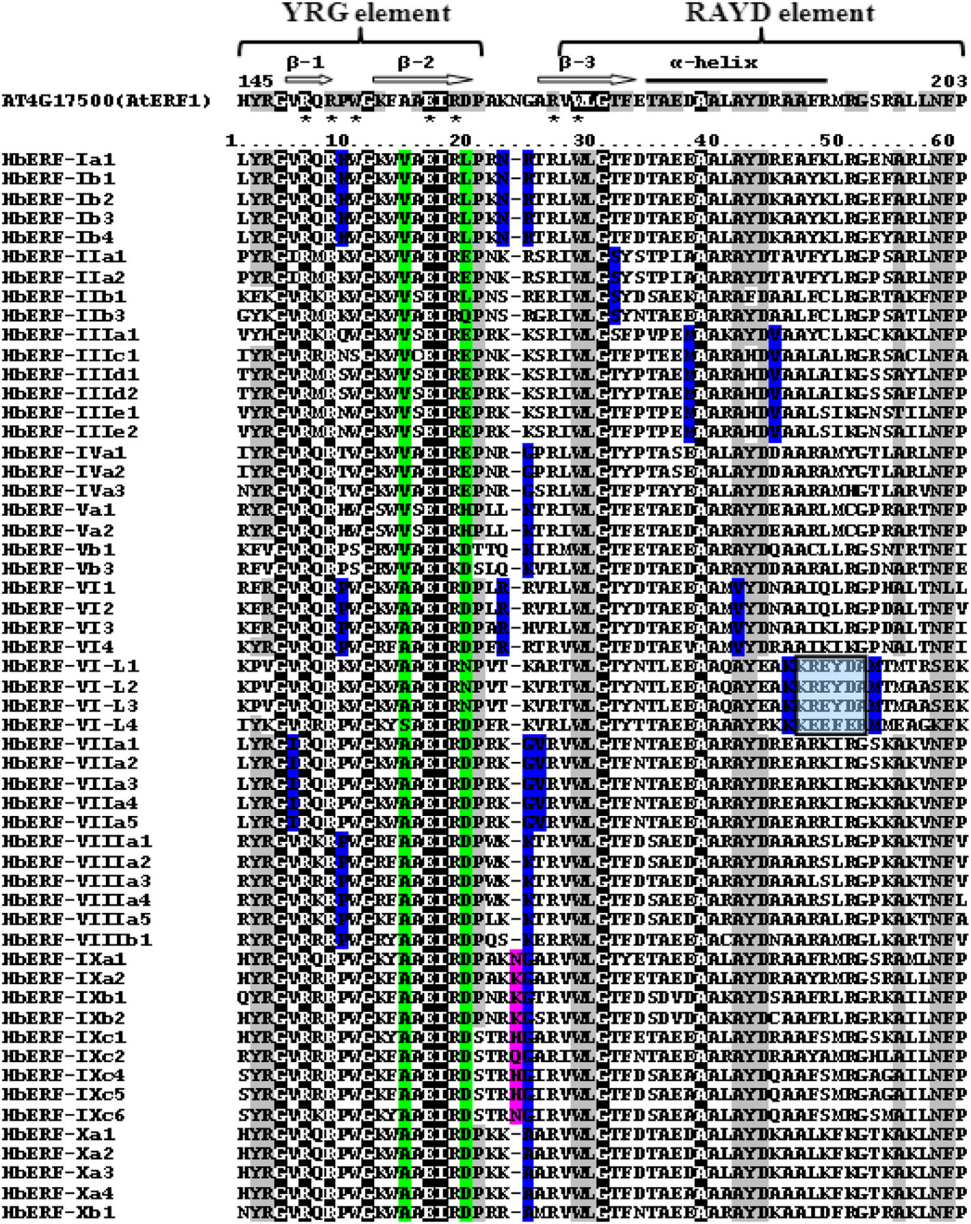
**Alignment of the AP2/ERF domains in *Hevea *(55 representative members).** Black and light grey shading indicate identical and conserved amino acid residues, respectively. Light blue shading indicates conserved amino acid residues in group VI-L. Green indicates the V14, E19 residue conserved (Yoh Sakuma,2002); blue indicates the residue conserved in each group individually; pink indicates the supplementary residue in group IX. The black bar and block arrows represent predicted a-helix and b-sheet regions, respectively, within the AP2/ERF domain (Allen et al., 1998). Asterisks represent amino acid residues that directly make contact with DNA (Allen et al., 1998). The YRG, RAYD elements are indicated according to (Okamuro, 1997).

The amino acid sequences of the AP2 domain from fifty-five representative *ERF* genes with full-length transcript sequences were aligned in order to identify the structure and the group-specific residues (Figure 4). Tertiary structures of the AP2 domain were predicted and revealed similarity to AtERF1 for each gene consisting of a three-stranded anti-parallel β-sheet and one α-helix (Protein Database number 2GCC). Specific amino acid residues were also identified for each group. AP2 domains from ERF family proteins contained the WLG motif and most of them also contained the YRG and RAYD elements. The positions of the AP2 domain were numbered according to the three-dimensional structure of AtERF1. Ten amino acids were totally conserved in each group (G148, R150, R152, G155, E160, I161, W172, L173, G174 and A182, Figure 4). Most AP2 domain sequences had conserved amino acid residues: V158 and E163 for groups I to IV and A158 and D163 for groups V to X, which corresponded to V14 and E19 for DREB and A14 and D19 for the ERF subfamilies according to Sakuma’s classification, respectively (Figure 4 and Additional file 2: Figure S1). A few members that did not show any conservation at these positions 158 and 163 were categorized based on their placement in the phylogenetic tree. A conservative sequence motif of 5 amino acid residues (KREYD) only occurred in group VI-L.

The group-specific amino acid residues observed in *Hevea* were compared with those of *Arabidopsis* and *Gossypium* (Table 7). At least one group-specific residue could be identified for each group, two for groups II and VIII, and three for group VII. *Hevea* group VI-L revealed one more group-specific residue (M196) in addition to the K189 found in all species. For group IX, one additional residue at position 167 was identified for all species leading to an AP2 domain of 59 amino acids long, as opposed to 58 for the other groups. In the *Hevea*, *Arabidopsis* and *Gossypium* AP2 family, the AP2 domains contain a conserved amino acid, T150 (92%) or A150 (8%). The AP2 domains of the RAV family have the amino acid residue V150 conserved at 100% in *Hevea*, *Arabidopsis* and *Gossypium*.

**Table 7:** Group-specific residues present in the AP2 domain representative of each family and each ERF group

**Family**	**Group**	**Group-specific residues**			**Conservation**
		***Arabidopsis***	***Gossypium***	***Hevea***	**(%)**
AP2		T150/A150	T150/A150	T150/A150	92-8
ERF	I	R168	R168	R168	100
	II	S175 -Y176	S175- Y176	S175 -Y176	97-97
	III	M181	M181	M181	100
	IV	G168	G168	G168	100
	V	K168	K168	K168	100
	VI	P153	P153	P153	100
	VI-L	K189	K189	K189 - M196*	100
	VII	I149 - G168 - V169	I149 - G168 - V169	I149 - G168 - V169	100-100-100
	VIII	P153 - K168	P153 - K168	P153 - K168	98-98
	IX	+X167	+X167	+X167	100
	X	A168	A168	A168	100
RAV		V150	V150	V150	100

The presented residues were the most conserved for the three compared species (*Hevea brasiliensis, Arabidopsis thaliana, Gossypium hirsutum*). (*) *Hevea*-specific residue compared to the other two species. (+) Additional amino acid residue compared to the other groups. (X) non-conserved residue.

### Distribution of reads from *AP2/ERF* contigs in the various tissue-type libraries

**Figure 5:**
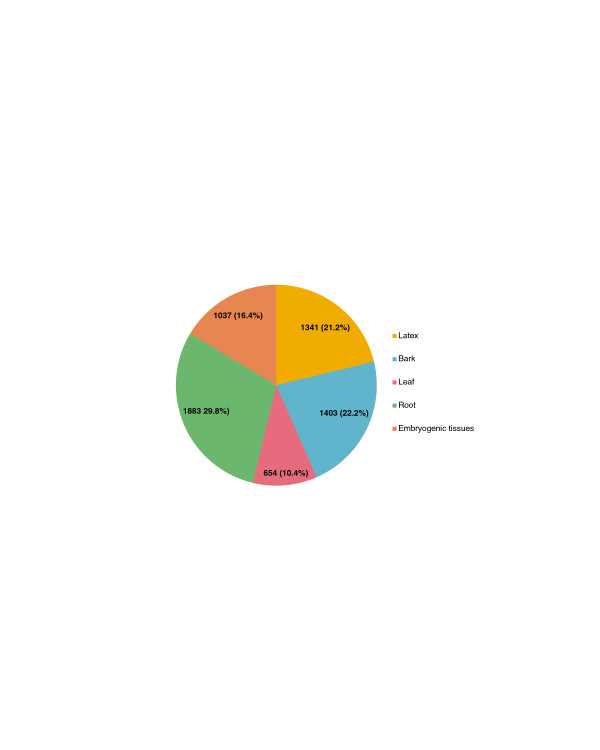
**Distribution of reads and percentage of total AP2/ERF contigs in the different tissue-type libraries (latex, bark, leaf, root, embryogenic tissues) in *Hevea*.**

The distribution of reads constituting AP2/ERF contigs in each tissue-type library reflected the global level of expression of *AP2/ERF* genes in each tissue (Figure 5). The number of reads was more abundant in roots with 29.8% (1883 reads), bark with 22.2% (1403 reads), followed by latex with 21.2% (1341 reads), embryogenic tissues with 16.4% (1037 reads) and then leaves with 10.4% (654 reads).

**Figure 6:**
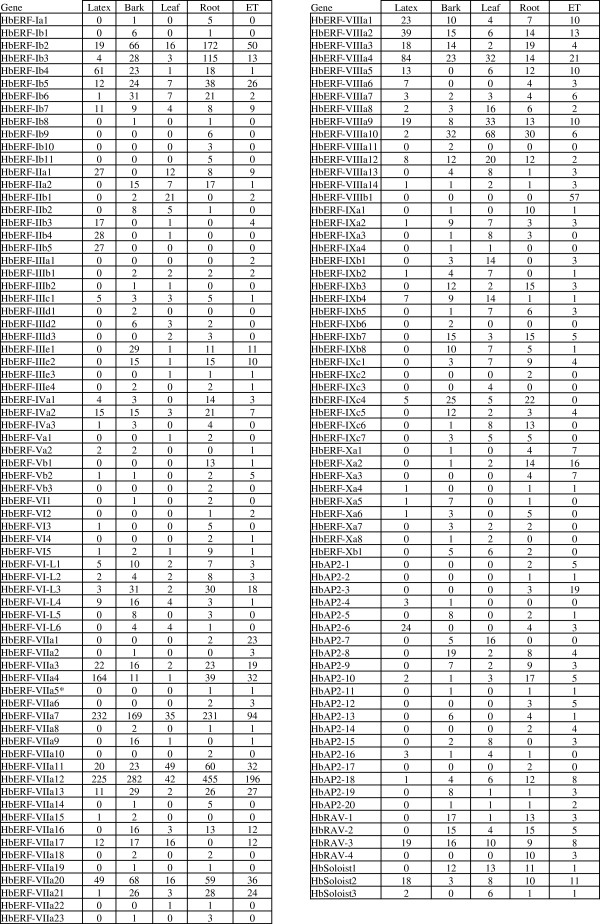
**Distribution of reads for each AP2/ERF contig in the different tissue-type libraries (latex, bark, leaf, root, embryogenic tissues (ET) in *Hevea*.**

The sum of reads for the various ERF groups showed that some groups were more represented in some tissues (Table 8). A higher read number was observed in latex for groups II, VII and VIII, in bark for groups III, VI-L and IX, in leaves for groups II, VIII and IX, in roots for groups I, IV, V, VI and VII, and in embryogenic tissues for group X only. The DESeq statistical analysis of the read distribution for each contig did not revealed any significant differential gene expression (Additional file 3; Figure 6). All AP2/ERF families and all ERF groups were represented. Thirty-seven transcripts were detected in all five tissues. Fifty-nine contigs were built from reads found in latex.

**Table 8:** **Distribution of reads for each ERF group in the various tissue-type libraries in*Hevea***

	**Total reads**	**Bark**	**Embryo**	**Latex**	**Leaf**	**Root**
**Group I**	829	189	101	108	38	393
**Group II**	213	25	16	99	47	26
**Group III**	148	60	28	5	14	41
**Group IV**	93	21	10	20	3	39
**Group V**	33	3	7	3	1	19
**Group VI**	29	3	4	2	1	19
**Group VL-L**	183	73	25	19	14	52
**Group VII**	3061	683	516	737	171	954
**Group VIII**	832	126	150	219	200	137
**Group IX**	368	112	29	14	101	112
**Group X**	100	21	31	3	12	33
**Total**	**5889**	**1316**	**917**	**1229**	**602**	**1825**

### Expression profile in various tissues for ten selected AP2/ERF genes

**Figure 7:**
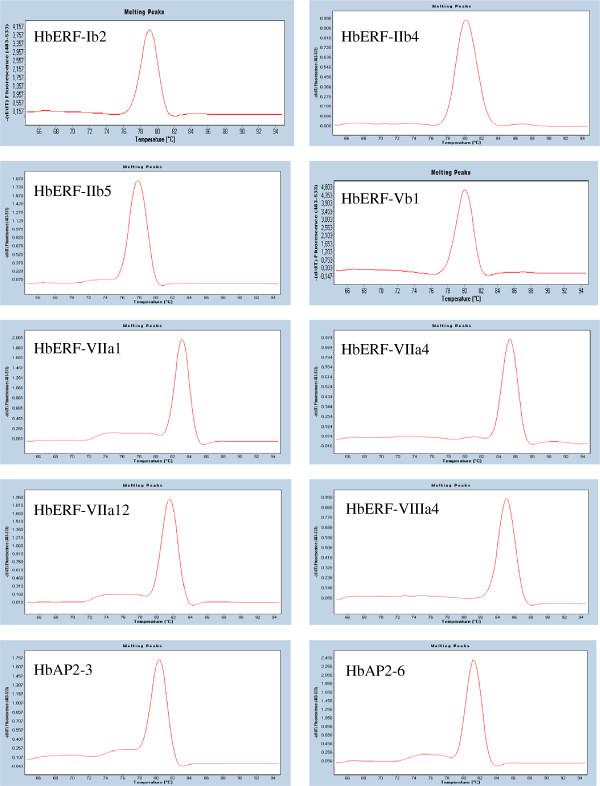
Analysis of the fusion curves after real-time RT-PCR amplification for ten AP2/ERF genes.

**Figure 8:**
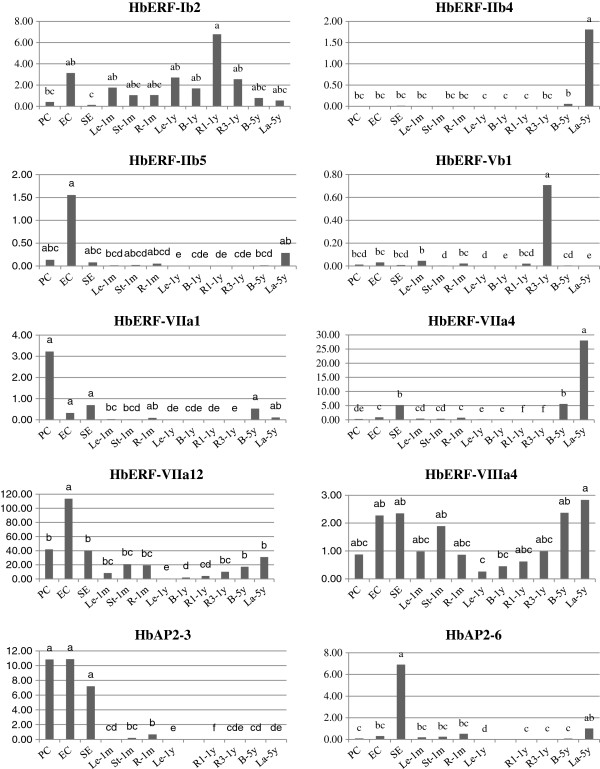
**Expression profile of 10 AP2/ERF genes selected for their contrasting distribution of reads for contigs in various tissues.** PC: proliferating callus grown on Maintainance Medium; EC: embryogenic callus grown on Expression Medium; SE: mature somatic embryos; Le-1 m: leaf of 1-month-old plantlets; St-1 m: stem of 1-month-old plantlets; R-1 m: root of 1-month-old plantlets; Le-1y: leaf of 1-year-old plants; B-1y: bark of 1-year-old plants; R1-1y: tap-root of 1-year-old plants; R3-1y: secondary lateral root of 1-year-old plants; B-5y: bark of 5-year-old trees; La-5y: latex of 5-year-old trees. Values are the means of the relative transcript abundance of three biological replicates. The data were analysed with XLSTAT software after log transformation. Statistical analysis was performed with an ANOVA after logarithmic transformation of raw data. The ANOVA was followed by a Student Newman-Keuls test. Values with the same letter did not differ significantly at the 0.05 probability level.

Ten *AP2/ERF* genes were selected for their high number of reads per contig or their presence in some tissue-type libraries. Primer were designed (Table 9), and their specificity was confirmed for each gene by a unique pick of the fusion curve after real-time RT-PCR amplification (Figure 7). Their relative transcript abundance was checked by real-time RT-PCR using *HbRH2b* as stable internal control between each tissue (Figure 8). The highest relative transcript abundance was found for *HbERF-VIIa12*, which ranged from 0.4 to 114. Interestingly, *HbERF-IIb4* and *HbERF-VIIa4* showed significant higher relative transcript abundance in latex than other tissues, 1.8 and 28 respectively, like it was observed for the read distribution. Nevertheless, latex specificity of *HbERF-IIb5* expression was not proved since transcripts of this gene were also highly accumulated in embryogenic callus. Relative transcript accumulation was noted in embryogenic tissues (proliferating callus, embryogenic callus or somatic embryos) for *HbERF-IIb5*, *HbERF-VIIa1*, *HbAP2-3* and *HbAP2-6*. The high read distribution counted in root was confirmed by high relative transcript abundance in the tap root of one-year-old plants. Finally, no significant difference could be found in relative transcript abundance for *HbERF-VIIIa4* in contrast with the higher read distribution in latex and leaf compared with other tissues.

**Table 9:** **List of primer sequences for 10 selected AP2/ERF genes from *H. brasiliensis *clone PB 260**

**Gene**	**Contig accession number in the global library**	**Primer sequences**	
		**Forward**	**Reverse**
HbERF-Ib2	hevea_454_rep_c703	CCACCAAGGAGCTCATGTCT	CTTCTCCTGTGAAGGAGCTGA
HbERF-IIb4	hevea_454_rep_c20637	CCTCCACCGGCTTCTACTATT	CCACCATCTCTTCTCTCTCCTC
HbERF-Vb1	hevea_454_rep_c20790	AGTAGCAGCAGCACGAGTGA	AGTCCAGCACCTCCTGAGAA
HbERF-VIIa1	hevea_454_rep_c16874	CGAGGAGAATTCTGGGTCTG	TCTGCACTTCGCTCTCTTGA
HbERF-VIIa4	hevea_454_rep_c1157	AGCAGGAGAGCGAAGTGCAGAA	AACAATTGCCGTCGCATCCACC
HbERF-VIIa12	hevea_454_rep_c110	AGATGAAGCCTGACTCTGGAA	CTCCACAGGTCCATTGGATT
HbERF-VIIIa4	hevea_454_rep_c2227	GCTGACAACAGCAACGGCAACA	TTCTGCAGCTCAAGGACGGTGA
HbERF-IXb7	hevea_454_rep_c9858	AAGGCAAGGCAGCTCAATC	ACCCAAACAAACCGTTATCC
HbAP2-3	hevea_454_c24965	TACTGCCGCAAACAACTGAC	CCTGTCTTTCTTGCCTGCTC
HbAP2-6	hevea_454_rep_c16078	AATGCCAGCGAGTTACCAAG	TGGTTGTCGAACAAGATGGA

### Prediction of microRNA-targeted *AP2/ERF* genes

Twelve microRNAs (miR156, miR159, miR172, miR393, miR395, miR396, miR408, miR894, miR1511, miRn11, miRn12, miRn14) were predicted to inhibit *Hevea* transcripts of twenty-nine *HbAP2/ERF* genes (Table 10). Eight microRNA families (miR159, miR172, miR393, miR408, miR894, miRn11, miRn12, miRn14) were predicted to inhibit thirteen latex-expressed transcripts (*HbAP2-4, HbAP2-6, HbAP2-10, HbAP2-18, HbERF-Ib5, HbERF-IIa1, HbERF-VI5, HbERF-VI-L1, HbERF-VIIa4, HbERF-VIIa13, HbERF-VIIa20, HbERF-VIIIa7, HbSoloist3*). Although inhibition mostly involved a transcript cleavage, the inhibition of translation was predicted for nine genes (*HbERF-Ib5, HbERF-IIa1, HbERF-VI5, HbERF-VIIa4, HbERF-VIIIa7, HbERF-IXc3, HbERF-IXc5, HbERF-Xa1, HbSoloist3*). Predicted microRNA sites were in the conserved AP2 domain (*HbERF-IIId3, HbERF-IXb1, HbERF-IXc6, HbERF-Xa1* and *HbERF-Xa8* genes), in the CDS outside the AP2 domain for sixteen genes, and in the UTR for eight genes.

**Table 10:** **List of putative targets of conserved miRNAs and their mode of inhibition predicted both by psRNATarget server (**http://plantgrn.noble.org/psRNATarget/**) and by MIRANDA included in the LeARN pipeline**

**microRNA family**		**Target gene**		**UPE**	**Free energy**	**miRNA size**	**miRNA aligned fragment**	**Target aligned fragment**	**Inhibition**	**MiR position**	**miR position binding with respect to CDS**
**Name**	**Accession No**	**Gene name**	**Contig accession No**			**No bases**				**bp**	
miR156	acc_480780	*HbAP2-9*	hevea_454_rep_c24306	24.478	−24.41	23	UGACAGAAGAGAGAGAGCACAUC	UACUCUCUUUUUUCUGCCAA	Cleavage	1001-1020	Inside CDS & after AP2 domain
miR159	acc_19665	*HbERF-IXc2*	hevea_454_c72747	13.691	−25.86	23	UUUUGAUUGAAGGGAGCUCUAAU	GAGCACCCUUCAAUUAAG	Cleavage	297-314	Inside CDS & after AP2 domain
miR159	acc_19665	*HbERF-VI-L1*	hevea_454_rep_c17780	16.359	−27.80	23	UUUUGAUUGAAGGGAGCUCUAAU	GUUCUAGCUUCCUUCAAGCAGAG	Cleavage	50-72	outside CDS, 5’UTR & before AP2 domain
miR172	acc_502684	*HbAP2-18*	hevea_454_rep_c22185	15.621	−21.57	21	UAGCAUCAUCAAGAUUUUUAU	AAGAGAAUCCUGAUGAUGCUG	Cleavage	1473-1493	Inside CDS & after AP2 domain
miR172	acc_502684	*HbAP2-20*	hevea_454_rep_c45080	17.625	−24.25	21	UAGCAUCAUCAAGAUUUUUAU	AUGAGAAUCCUGAUGAUGCUG	Cleavage	990-1010	Inside CDS & after AP2 domain
miR393	acc_112860	*HbAP2-4*	hevea_454_c60993	24.258	−22.79	25	UUCCAAAGGGAUCGCAUUGAUUAUC	AGCAAUGUUAUUCCUUUGGC	Cleavage	198-217	Inside CDS & before AP2 domain
miR395	acc_262739	*HbERF-IXc3*	hevea_454_c37716	15.053	−24.47	25	CUGAAGUGUUUGGGGGACCUCAUC	GAGAAAGUUCUCCAAUCACUUCAA	Translation	243-266	Inside CDS & after AP2 domain
miR396	acc_7978	*HbRAV-2*	hevea_454_rep_c13430	22.097	−22.40	24	CCACAGCUUUCUUGAACUGCAAUC	GAGUUCAAGAAAGCGGUU	Cleavage	562-579	Inside CDS & after AP2 domain
miR408	acc_135004	*HbERF-IXb1*	hevea_454_c13287	14.501	−23.60	23	UGCACUGCCUCUUCCCUGCCAUC	AAGAGAAGAGGCAGUACA	Cleavage	118-135	Inside CDS & cut AP2 domain
miR408	acc_184014	*HbERF-VIIa9*	hevea_454_rep_c64305	24.62	−28.45	24	UACACUGCCUCUUCCCUGGCUAUC	UGCGAGCAGGAGGAGAGGCAGU	Cleavage	207-228	Inside CDS & before AP2 domain
miR408	acc_184014	*HbERF-VIIa13*	hevea_454_rep_c8113	24.62	−28.45	24	UACACUGCCUCUUCCCUGGCUAUC	UGCGAGCAGGAGGAGAGGCAGU	Cleavage	518-539	Inside CDS & before AP2 domain
miR894	S_seq_24257	HbERF-IIa2	hevea_454_rep_c7563	15,655	−19,42	16	UGUUUCACGUCGGGUU	AACCUGACUUGAACCA	Cleavage	760-775	Inside CDS & after AP2 domain
miR894	S_seq_73030	HbAP2-6	hevea_454_rep_c16078	18,933	−22,38	17	CGUUUAACGUCGGGUUC	GAAGCCGAUGUUGAACA	Cleavage	694-710	3’ region & after CDS
miR894	S_seq_174443	HbERF-IIIe3	hevea_454_rep_c58258	21,025	−23,87	20	UCUUCGUUUCACGUCGGGUU	AAUCUGCCGUGGUACGAGGA	Cleavage	653-672	Inside CDS & after AP2 domain
miR894	S_seq_181990	HbERF-IXc5	hevea_454_rep_c36947	23,707	−25,35	20	GUUUCACAUCGGGUUCACCA	UGGAGAAAUCCAUGUGAAAU	Translation	901-920	5’ region & before CDS
miR894	S_seq_376904	HbERF-VIIa20	hevea_454_rep_c710	17,302	−27	16	GUGCGUUUCACGUCGG	UUGCCGUGAAACGCAU	Cleavage	130-145	Inside CDS & before AP2 domain
miR894	S_seq_376904	HbERF-VIIa23	hevea_454_rep_c88669	17,295	−27	16	GUGCGUUUCACGUCGG	UUGCCGUGAAACGCAU	Cleavage	73-88	Inside CDS & before AP2 domain
miR1511	S_seq_556556	HbERF-IXc6	hevea_454_rep_c18341	22,461	−27,04	19	AACCAGGCUCUGAUACCAU	AUGGCAUCAGGGUUUGGUU	Cleavage	449-467	Inside CDS & inside AP2 domain
miRn11	S_seq_275942	HbERF-VIIa2	hevea_454_rep_c40731	16,586	−26,87	16	AGUUUGGCUGGGGCGG	UCGCCUUAGCUAAACA	Cleavage	37-52	5’ region & before CDS
miRn11	S_seq_275942	HbERF-VIIIa7	hevea_454_rep_c16802	16,135	−24,03	19	AGUUUGGCUGGGGCGGCAC	GCGCAGCCCAAGCCAAACC	Translation	355-373	Inside CDS & after AP2 domain
miRn11	S_seq_11884	HbERF-IIId3	hevea_454_c39573	13,87	−31,03	16	GGGAGUUUGGCUGGGG	UCCUAGCCAAAUUCUU	Cleavage	405-420	Inside CDS & inside AP2 domain
miRn11	S_seq_11884	HbERF-Xa8	hevea_454_c51284	21,466	−28,52	15	GGGAGUUUGGCUGGG	CCUAGCCAAACUCUA	Cleavage	419-433	Inside CDS & Inside AP2 domain
miRn11	S_seq_4512	HbERF-VI5	hevea_454_c22933	4,586	−24,23	17	GAGUUUGGCUGGGGCGG	UCGUCCAAGCCCAACUC	Translation	215-231	5’ region & before CDS
miRn11	S_seq_422499	HbERF-VIIa4	hevea_454_rep_c1157	11,652	−23,53	16	GAGUUUGGCUGGGGCU	AGUCCUAUCCGAACUC	Translation	457-472	Inside CDS & before AP2 domain
miRn11	S_seq_234287	HbSoloist3	hevea_454_rep_c46638	20,623	−37,87	19	UCAGGUGGG-GAGUUUGGCU	AGCCAGACUCAUCCACCUGA	Translation	618-637	Inside CDS & after AP2 domain
miRn12	S_seq_119244	HbERF-IIa1	hevea_454_rep_c8625	11,7	−21,45	16	UUUGCAGUUCGAAAGU	AUUUUUUAACUGUAAG	Translation	982-997	5’ region & before CDS
miRn12	S_seq_7691	HbAP2-10	hevea_454_rep_c22185	23,384	−26,47	17	UUAGCAGUUCGAAAGUG	CACCCUUGAACUGCUAA	Cleavage	39-55	3’ region & after CDS
miRn12	S_seq_2869	HbERF-Xa1	hevea_454_c58761	22,067	−32,35	17	UUGGCAGU-UCGAAAGUG	UACUUUCGAUACUGCCGA	Translation	611-628	Inside CDS & inside AP2 domain
miRn14	S_seq_358709	HbERF-Ib5	hevea_454_rep_c4396	8,491	−24	26	UAGAACACAAUUAUAGGAAUCAAUAU	AUAUUGGU-CUUGUACUUGUGUUCUG	Translation	1936-1960	3’ region & after CDS

Target accessibility is represented as the maximum energy needed (UPE) to unpair the secondary structure around target site on target mRNA. The lower the energy the greater the possibility that small RNA is able to contact (and cleave) target mRNA. The lower the free energy the greater the possibility that small RNA is able to contact target mRNA. The predicted microRNA was based on a free energy threshold below −20 kcal/mol.

## Discussion

### NGS data combined with an optimized method of alignment and classification led to the identification of the *Hevea* AP2/ERF superfamily

The AP2/ERF superfamily has been identified in several species from both genome and EST sequences. For the first time to our knowledge, this study presented the identification of most members of the AP2/ERF superfamily using the 454 sequencing technology for crop plants for which few data are available and especially for rubber. The one hundred and seventy-three *AP2/ERF* members identified in *Hevea* were clustered into three main families (25 AP2, 141 ERF, and 4 RAV members) and a group of 3 soloists using a maximum likelihood phylogenetic analysis. The ERF family was then subdivided into 11 major groups, which corresponded to groups I to X, and group VI-like described by Nakano [27]. The stringency used for the read assembly led to discriminate the various allelic forms. *Hevea brasiliensis* is highly heterozygous that should lead to have various allelic forms in the assembled contigs and consequently less genes than *AP2/ERF* members. The number of *Hevea AP2/ERF* genes was comparable to the number observed in other species. RNA sequencing of additional tissues, such as flowers, should lead to cover the whole transcriptome.

The first phylogenetic analyses came up against the low quality of contig sequences from NGS (data not shown). The minimum overlap length was increased to 60 bp compared to the 40 bp used in *Jatropha curcas* for instance, with a minimum overlap identity of 95% [57]. Finally, the assembly strategy for *Hevea* reads delivered robust contigs from current programs since the clustering method discriminated conserved domains from the various *AP2/ERF* genes. Sequences of AP2/ERF genes were shown to be from unique transcript for 10 genes in this study and more recently for 132 genes by analysis of the fusion curve after real-time RT-PCR amplification (data not shown). In addition, homopolymer correction by mapping Solexa reads was not required. The Neighbour-Joining tree built from the protein distance matrix with manual correction proposed by Nakano was widely adopted for classification of the ERF family. Based on NGS contigs, the classification method proposed by Nakano provided inconsistent results due to errors and the accuracy rate of contig sequences. An AP2 domain block of 57 amino acids was selected for the alignment of 142 sequences with a full AP2 domain using a combination of MUSCLE and Gblock softwares. The use of Gblocks reduced the need for manual editing of multiple alignments. This method facilitated the construction of a consistent phylogenetic tree with PhyML software without requiring a Bayesian Inference method. The latter method was successfully used to classify the *Arabidopsis* ERF protein family [58]. These authors included groups VI-like and Xb-like described by Nakano *et al.* in their phylogenetic reconstruction, and ultimately assigned these groups as new groups XI and XII, respectively. Group VI-L genes were close to group VI, with a modification in the second element suggesting that the evolution of group VI-L is more recent than that of the other groups. This independent cluster on the *Hevea* phylogenetic tree led us to propose it as a new group (see below).

### *Hevea AP2/ERF* genes have common and several specific features compared to other species

Several functionally important conserved motifs described in *Arabidopsis* and tomato were also found in *Hevea* AP2/ERF deduced proteins suggesting that they are likely to function as transcription factors [56]. The putative nuclear localization signal (NLS) motif near the R1 domain was found in *Hevea* AP2/ERF transcription factor sequences (data not shown). The residues G148, R150, R152, G155, E160, I161, G174 and A182 were completely conserved among all 437 ERF proteins collected from three species (*Hevea, Arabidopsis* and *Gossypium*). These observations are generally consistent with earlier reports on this topic [27,34,39]. The conserved Ala-37 (corresponding to A182 in this paper) in the ERF domain has been suggested to play a major role in the stability of the ERF domain or DNA binding with the DRE element or GCC box [56,59]. The ERF-associated amphiphilic repression (EAR) motif was first described by Ohta [60]. The EAR motif is found in group II and VIII. DEAR1, a DREB protein-containing EAR motif, has been shown to mediate crosstalk between signalling pathways for biotic and abiotic stress responses [61]. The EAR motif exists in all members of ERF group VIII in tomato [56] and in ERF group VIIIa in *Arabidopsis*[27,28,45].

Soloists have been characterized by low conservation at the ERF DNA-binding domain in all plant genomes considered [45]. In *Hevea*, we showed that this low conservation could be explained by 6 missing amino acid residues in their AP2 domain, including R152, which directly contacts the GCC box [62]. The three *HbSoloist* genes only shared between 65% and 73% identity in their nucleotide sequences, which led us to consider these as three different *HbSoloist* genes and not as allelic forms. Although the three *HbSoloist* genes have only a single AP2 domain, they formed a group and clustered together with the AP2 family, as has been reported in *Vitis vinifera*[45]. The greater conservation in amino acid sequences than in nucleotides, especially for the AP2 domain, revealed an evolutionary constraint suggesting a putative function for *Hevea* soloists since there were recent duplications. However, no functional information has been published for soloist genes.

Based on an analysis of 437 AP2 domain sequences of ERF genes from three species, ten amino acid residues were shown to be strictly group-specific for all ERF groups except for group II and group VIII. A previous study on 315 AP2 domain sequences from *Arabidopsis*, *Gossypium* and *Oryza* led to the identification of 14 group-specific residues with a certain error rate [48]. The group-specific residues reported in this study could be proposed as a group marker of the ERF family for several species. In addition, *Hevea AP2/ERF* genes harboured unique group-specific residues in their AP2 domain, such as VI-L (M196), which are not found in other species. This difference could be explained by the distance between *Gossypium* and *Arabidopsis* in the Eurosides II (Brassicales and Malvales, respectively) and *Hevea* in the Eurosides I (Malpighiales) [63]. We also identified that position 150 was conserved in *Hevea*, *Arabidopsis*, *Gossypium*, *Populus* with T150, T150 and V150 for the ERF, AP2 and RAV families, respectively. Position 150 directly contacts with DNA. These interactions determine the geometry of the GCC-box binding domain (GBD) relative to DNA and thereby comprise a framework for specific base recognition [62].

### Several *AP2/ERF* genes highly expressed in latex could be related to a specific function in *Hevea*

*AP2/ERF* genes are regulated by developmental processes and environmental cues [64]. As rubber trees are subjected to frequent mechanical wounding and osmotic stress upon tapping to collect latex, and ethephon stimulation to increase latex yield, some of these transcription factors are likely to play a unique role in *Hevea* defence mechanisms and latex production. Latex cells are differentiated in phloem tissue from cambium [10]. Members of the AP2 family play an important role in angiosperm reproductive organ development [65-68]. Members of the RAV family were reported to be induced in ethylene response and in brassinosteroid response and to be involved in flower senescence [69]. Consequently, the AP2 and RAV genes present in latex are suggested to play an important role in *Hevea* development.

Several of the fifty-one ERF transcripts accumulated in latex could be related to responses to stress. High read abundance was found in latex for ERF groups II, VII and VIII. Latex cells are differentiated in roots, leaves and bark. This might explain why genes expressed in latex could also be identified in the other tissues. In addition, thirteen other transcripts were highly accumulated in latex compared with other tissues: one for the AP2 family, one for the RAV family and eleven for the ERF family. The ERF transcripts highly accumulated in latex were distributed as follows: one for group I, four for group II, two for group VII and four for group VIII. Large number of genes was identified for groups VII, VIII and IX with 23, 15 and 19 genes, respectively.

A few members of the AP2/ERF superfamily have been previously reported in *Hevea*. The *HbERF1*, *HbERF2*, *HbERF3* and *HbRAV1* genes were suggested to be induced by JA in bark during JA-induced laticifer differentiation [70]. According to our analysis, the *HbERF1*, *HbERF2* and *HbERF3* genes corresponded to *HbERF-VIIa3*, *HbERF-VIIa17* and *HbERF-VIIa1* in our classification with 99%, 98%, 99% identity, respectively. The *HbCBF1* gene [71], and the *HbCBF2* gene [5] have been reported to be regulated by cold and drought stresses, like other members of the DREB subfamily. We classified these genes in ERF group III. The *HbCBF1* gene corresponded to the *HbERF-IIIc1* gene with an identity of 100%, and the *HbCBF2* gene to the *HbERF-IIIb2* gene with 82% identity. Another member of the AP2/ERF superfamily is the *HbEREBP1* gene recently identified by Chen *et al.* from *Hevea* laticifers [72]. This gene was down-regulated by tapping and mechanical wounding in laticifers from adult trees, and was also regulated by both exogenous ethephon or methyl jasmonate treatments. This suggests that the *HbEREBP1* gene may be a negative regulator of defence mechanisms in laticifers [72]. The *HbEREBP1* gene corresponded to the *HbERF-VIIIa12* gene with 100% identity in our analysis.

### Eleven new microRNAs are predicted to inhibit *Hevea* AP2/ERF transcripts

The mode of action of miR172-regulated *AP2* genes has been well described in reproductive and vegetative organs as well as in the transition of developmental phases [73,74], where multiple feedback loops involve the microRNAs miR156e targeting Squamosa Promoter Binding Protein-like (SPL) and miR172b targeting AP2 [75]. Seven gymnosperm AP2 homologs were found to contain a sequence corresponding to miR172 with an average identity of approximately 84.4%, suggesting that mechanisms regulating gene expression using microRNAs have been conserved over the three hundred million years since the divergence of gymnosperm and flowering plant lineages [76]. The cleavage site of miR172 is conserved between plant lineages and is located between the second AP2 domain and the 3′ terminus [76]. This site is also observed in *Hevea*. However, miR172 regulates flowering time by down-regulating *AP2-like* target genes by a translational mechanism rather than by RNA cleavage [74], and could explain our failure in detecting cleaved *HbAP2-18* and *HbAP2-20* transcripts (data not shown). In addition to miR172, eleven other microRNAs were predicted to inhibit *Hevea* transcripts of twenty-nine *HbAP2/ERF* genes. Seven microRNA families were only found in various tissues of plantlets [77], and five others only in the latex of mature trees, including three novel microRNA families (miRn11, miRn12, miRn14) (data not shown). For the first time to our knowledge, both cleavage and translation inhibition were predicted with miR binding in the CDS sequence and especially for 5 genes in the AP2 domain.

## Conclusions

NGS sequencing of five tissue-type libraries led to the generation of transcriptome data from the broadest coverage of tissues in *Hevea* compared with previous work done on latex, leaf and bark [49,50,78,79]. This allowed identifying 173 AP2 domain-containing transcripts, of which 142 had a full-length AP2. We have proposed an optimized alignment and classification method enabling the use of NGS data with repeatable outputs. Analysis of read abundance led to the prediction that ERF genes play a major role in laticifers. A comparison with *Populus* and *Vitis* did not provide any specific features for woody species as assumed earlier [45], but the AP2 family appeared to be well represented for these species. Several *AP2/ERF* genes highly expressed in latex could be related to a specific function in *Hevea.* Further studies focusing on latex cells should provide a clearer understanding of the involvement of genes from the AP2/ERF superfamily in the regulation of latex production and latex cell differentiation. Finally, analysis of allelic variations between transcript sequences of several *Heve*a clones could be useful for the development of functional molecular markers.

## Methods

### Plant material

Plant material of clone PB 260 was grown according to the conditions described in the Table 1. Self-rooted plants were produced by somatic embryogenesis at the CIRAD laboratory [55,80]. Total mRNAs were isolated from different tissues. The embryogenic tissue sample was a mix of proliferating callus, embryogenic callus and somatic embryos. Leaf, root and bark tissues were taken from *in vitro* plantlets and grown for up to 1 month and 1 year after acclimatization. At each time point, *in vitro* plants were treated for 4 and 24 h with 1 ppm of ethylene or by wounding, or by water deficiency up to wilting leaves [14,81]. Leaf, root and bark tissues were also taken from three-month-old shoots from grafted plants treated by wounding and 1 ppm of ethylene. The leaves were mechanically wounded by squeezing the entire surface of the leaves with pincers whilst the bark was wounded every 0.5 cm by scarification with a razor blade. Latex and bark samples were taken at IRRI’s Sembawa Centre from 5-year-old trees that were either untapped, tapped or both tapped and stimulated with 2.5% ethephon before RNA isolation.

### Total RNA isolation

Leaves, bark, roots, embryogenic tissues (somatic embryos and callus) were frozen in liquid nitrogen and stored in the freezer at -80°C pending total RNA extraction. Total RNA was extracted using the caesium chloride cushion method adapted from Sambrook [82] by Duan and coll. [14]. One gram of fresh matter was ground and transferred to a tube containing 30 ml of extraction buffer consisting of 4 M guanidium isothiocyanate, 1% sarcosine, 1% polyvinylpyrrolidone and 1% ß-mercapto-ethanol. After homogenization, tubes were kept on ice and then centrifuged at 10,000 g at 4°C for 30 minutes. The supernatant was transferred to a new tube containing 8 ml of 5.7 M CsCl. Ultracentrifugation in a swinging bucket was carried out at 115,700 g at 20°C for 20 hours. The supernatant and caesium cushion were discarded whilst the RNA pellet was washed with 70% ethanol. After 30 minutes of air drying, the pellet was dissolved in 200 μl of sterile water. RNAs were conserved at −80°C.

The procedure for total RNA isolation from latex was derived from the method described by Kush et al. [83]. Six ml of latex was mixed with 6 ml of 2X alkaline fixing buffer (0.1 M Tris–HCl, 0.3 M LiCl, 10 mM EDTA, 10% SDS pH 9) and immediately deep-frozen in liquid nitrogen for storage. The mixture was then thawed and centrifuged for 30 min at 15,000 g. The aqueous fraction was treated with a phenol:chloroform solution twice, including centrifugation for 15 min at 10,000 g at 4°C. RNAs were precipitated overnight at 4°C after the addition of 1/3 volume of 8 M LiCl to the aqueous phase. After centrifugation for 30 min at 10,000 g at 4°C, the RNA pellet was resuspended in 400 uL of DEPC water on ice and then treated with a phenol:chloroform solution twice. The RNAs were finally precipitated with a 1/10 volume of 3 M Na acetate, pH 5.2, and 3 volumes of absolute ethanol. After centrifugation, the RNA pellet was resuspended and the solution kept at −80°C.

### Sequencing technique and contig assembly

Total RNA samples of each tissue from plants grown under the various conditions were pooled together to generate five transcript libraries (embryogenic tissues, leaf, bark, latex and root). Single-stranded cDNA was synthesised from pooled RNA samples. Pyrosequencing was carried out using GS FLX (Roche / 454) Titanium run (Roche Applied Science) by the GATC-Biotech company in Germany. A 454 sequencing half-run (200 Mbp) generated more than 500,000 reads with an average read length of 400 bp for each library according to the manufacturer. Reads were analysed using the ESTtik tool (Expressed Sequence Tag Treatment and investigation kit) [84] modified for the analysis of 454 data (Table 2). Reads were first cleaned to avoid mis-assembly by discarding sequences that were both lower than 120 bp and of low complexity. We then discarded non-coding reads by comparing the reads against the fRNAdb database using the Megablast algorithm with an e-value cutoff of 1e-20 [85]. More than 400,000 cleaned reads were obtained for each library. Reads were then assembled in contigs using the TGICL program [86], integrated in the ESTtik pipeline (Figure 1). The removal of poor end regions of reads and the computation of overlaps between reads has been done using default parameters of CAP3 program (best hit cut-off for difference -b = 20; best hit for clipping –c = 12) [87]. Clustering was carried out for reads with an overlap of at least 60 bp and 94% identity between reads.

The second step was an assembly of reads from each cluster with greater stringency: the length of sequence overlap was then 60 bp with 95% identity between reads. The transcript sequence database consisted of contigs. An automatic annotation of each contig was attempted using the BLAST algorithm to find similar sequences using the *Arabidopsis thaliana* peptide database Tair9, the Uniprot databases Swissprot and TrEMBL, the non-redundant protein sequence database NR and the nucleotide sequence database NT from GenBank. Contigs were then annotated with Gene Ontology terms using Blast2GO on our Blast results [88]. We predicted peptide sequences for each contig using the prot4EST pipeline [89]. The peptide sequences were then annotated comparing the sequences on the InterPro signature database using the InterProScan web service [90]. A first assembly set was generated from reads of each tissue separately to create tissue-type transcript databases. The reads of all five tissue-type libraries were then re-assembled to generate one global transcript sequence database for *Hevea* clone PB260, subsequently called the global database. Contig sequences of the global library are available on CIRAD’s website http://esttik.cirad.fr/ and in public databases.

### Identification of AP2 domain-containing contigs

Firstly, we downloaded the AP2 domain of the 147 *Arabidopsis thaliana AP2/ERF* genes from the *Arabidopsis* Transcription Factor Database (ArabTFDB) (http://plntfdb.bio.uni-potsdam.de/v3.0/). BLASTX was carried out using the 147 AtAP2 domain amino acid sequences as protein subjects and nucleic acid sequences of contigs assembled in the HbPB260 transcript database as the query [91]. Conversely, TBLASTN was carried out using nucleic acid sequences of contigs as the subject and the 147 AtAP2 domain amino acid sequences as the query. The two BLAST files were combined in order to keep information obtained in both BLASTX analyses. Contigs were translated using prot4EST [92] or FrameDP [93] and AP2 domain-containing contigs were then identified using the Interpro database (IPR001471) [90]. This analysis was validated with the Conserved Domain Database (CDD) and Resource Group on NCBI [94]. The method led to the identification of contigs with a full and partial AP2 domain.

### Phylogenetic analysis of the AP2 domain from putative *AP2/ERF* genes

A multiple alignment analysis was performed on full-length AP2 domain sequences from *Hevea, Arabidopsis* and *Gossypium*. Phylogenetic trees were firstly generated with the Neighbour-Joining method for *Hevea, Arabidopsis* and *Gossypium* in order to classify the groups (data not shown). The full AP2-domain sequences derived from 142 *H. brasiliensis* AP2-domain proteins of around 60 amino acids were then aligned using MUSCLE software [43,95], which uses a progressive multiple alignment method. The alignment was curated by Gblocks software [96], searching for at least 10-amino-acid-long conserved blocks, and the block with 57 amino acids was extracted. This block of 57 amino acids was used to construct the phylogenetic tree using PhyML software [40], which implements a maximum likelihood tree reconstruction method, using the LG + gamma model, starting from a BioNJ tree [97]. The tree was drawn and displayed with the Dendroscope program, and rooted on the branch separating the AP2 and RAV family from the rest of the tree. Branch supports were computed using the aLRT-SHlike method, and those under 0.70 were discarded. For genes of the AP2 family having two AP2 domains, the sequence of the first AP2 domain (repeat-1 or R1) was preferentially selected for alignment. For three partial transcripts, the second AP2 domain (repeat-2 or R2) was chosen for alignment instead of the first, which is not present.

### Comparison of the classification between various species

Genes from the AP2/ERF superfamily are listed in Tables 3, 5 and 6 from publications on *Arabidopsis thaliana*[27]*, Populus trichicarpa*[28], *Vitis vinifera*[45], *Solanum lycopersicum*[56], *Gossypium hirsutum*[48] and *Triticum aestivum*[26]. For *Hevea brasiliensis,* the classification of the AP2/ERF superfamily was based on the phylogenetic analysis presented in this paper. In addition to data from the phylogenetic analysis, contigs corresponding to partial transcripts harbouring either a partial AP2 domain sequence or only one AP2 domain instead of two for genes of the AP2 family are included in the presentation of Table 3.

### Identification of conserved motifs and specific amino acid residues

AP2 domain amino acid sequences from the *Hevea* ERF genes were aligned using CLUSTALX. Conserved residues observed in *Hevea* sequences were compared with those of other species such as *Gossypium* and *Arabidopsis* in order to identify ERF group-specific residues [48,62].

### Extraction of read data from AP2/ERF contigs of each library and statistical analysis

Perl script was used to parse the alignment .ace file provided by the TGICL assembler in order to count the number of reads for each transcript and to identify the number of reads from each tissue (bark, leaf, latex, root and embryogenic tissues). The data are presented in Figure 6. Statistical analysis of differentially expressed genes was carried out using DESeq (v1.10.1) package in R software [98,99]. Firstly, we have estimated the effective library size. Secondly, the estimated dispersion for all transcripts are fitted using “blind” method, “fit-only” sharing mode and “local” fitType as parameter for the “estimateDispersions” function. Then, we performed the “nbinomTest” to get p-values. The p-values were adjusted for multiple testing using the Benjamini and Hochberg as proposed in the DEseq package.

### Prediction of microRNA-targeted *AP2/ERF* genes

Deep sequencing of *Hevea* was performed with Solexa/Illumina technology and led to the identification of miRNA sequences conserved between plant species and putative novel miRNAs specific to *Hevea*[77] using the LeARN pipeline [100]. The AP2/ERF sequences from *Hevea* were scanned with conserved and non-conserved miRNA sequences using both psRNATarget server (http://plantgrn.noble.org/psRNATarget/, [101] and MIRANDA, which is included in the LeARN pipeline [100] with custom parameters (gap_value = 2, mm_value = 1, gu_value = 0.5, score_threshold = 3, min_length_alignment = 15 and no_mismatch_positions = 10;11). Only the miRNA/target pairs displaying a free energy below −20 kcal/mol are presented in Table 10.

### Analysis of transcript abundances by real-time RT-PCR

Several rules were applied in order to reduce the risk of error in relative gene expression data. The integrity of total RNA was checked by electrophoresis. Primers were designed at the 3’ side of each sequence in order to reduce the risk of error due to short cDNA synthesis using the Primer 3 module of Geneious (Biomatters Ltd., New Zealand). Real-time PCR amplification and the fusion curve were carried out using a mix of cDNAs in order to check the specificity of each pair of primers. Primer sequences are listed for 10 selected genes according to their distribution of reads per contigs in Table 9.

cDNAs were synthesized from 2 μg of total RNA to the final 20 μL reaction mixture using a RevertAidTM M-MuLV Reverse Transcriptase (RT) kit according to the manufacturer’s instructions (MBI, Fermentas, Canada). Full-length cDNA synthesis was checked on each cDNA sample by PCR amplification of the Actin cDNA using primers at the cDNA ends. Quantitative gene expression analysis was finally carried out by real-time RT-PCR using a Light Cycler 480 (Roche, Switzerland). Real-time PCR reaction mixtures consisted of 2 μL RT product cDNA, 0.6 μL of 5 μM of each primer, and 3 μL 2 × SYBR green PCR master mix (LightCycler® 480 SYBR Green I Master, Roche Applied Sciences) in a 6-μL volume. PCR cycling conditions comprised one denaturation cycle at 95°C for 5 min, followed by 45 amplification cycles (95°C for 20 s, 60°C for 15 s, and 72°C for 20s). Expression analysis was performed in a 384-well plate. Samples were loaded using an automation workstation (Biomek NX, Beckman Coulter).

Real-time PCR was previously carried out for eleven housekeeping genes in order to select the most stable gene as the internal control for all compared tissues (*HbelF1Aa, HbUBC4, HbUBC2b, HbYLS8, HbRH2b, HbRH8, HbUBC2a, HbalphaTub, Hb40S, HbUbi, HbActin*) (Data not shown). *HbRH2b* was selected as the best reference gene according to its stability in the various tissues. The *HbRH2b* gene was amplified in each reaction plate in parallel with target genes. The transcript abundance level for each gene was relatively quantified by normalization with the transcript abundance of the reference *HbRH2b* gene. Relative transcript abundance took into account primer efficiencies. All the normalized ratios corresponding to transcript accumulation were calculated automatically by Light Cycler Software version 1.5.0 provided by the manufacturer using the following calculation: Normalized Ratio = 2 ^-Δ*(Cp target-Cp RH2b)*^.

Real-time PCR reactions were set up with three biological replications. Statistical analysis was performed with an ANOVA after logarithmic transformation of raw data. The ANOVA was followed by a Student Newman-Keuls test. Values with the same letter did not differ significantly at the 0.05 probability level.

## Abbreviations

AIL: AIntegumenta-Like; ANT: AINTEGUMENTA; AP2: APETALA2; CBF: Cold responsive element binding factor; DREB: Drought responsive element binding protein; ERF: Ethylene responsive factor; PCR: Polymerase chain reaction; RAP2: Related to APETALA2; RAV: Related to ABI3/VP1; RT: Reverse transcriptase.

## Competing interests

The authors declare that they have no competing interests.

## Authors’ contributions

XA and MS carried out contig assembly and generated the transcript sequence database. CD and JFD carried out the phylogenetic analysis. CD and AC studied the features of the AP2 domain. K carried out field experiments. K and MR carried out RNA isolations. PP carried out design of primers and real-time RT-PCR analyses. VG and JL identified microRNAs and their target genes. JP carried out the DESeq statistical analysis. PM and CD planned the experiments. PM, CD, JFD and MS participated in drafting the manuscript. All the authors read and approved the final manuscript.

## Supplementary Material

Additional file 1 Amino acid sequences of partial AP2 domain from the 45 AP2/ERF members found in Xia et al. (2011). Click here for file

Additional file 2: Figure S1 Alignment of the AP2/ERF domains from *H.brasiliensis* 115 ERF family proteins. Black and light gray shading indicate identical and conserved amino acid residues, respectively. Dark gray shading indicates conserved amino acid residues in group VI-L. Green color indicates the V14, E19 residue conserved [39]; blue color indicates the residue conserved in each group individually;pink color indicates the supplementary residue in group IX The black bar and block arrows represent predicted a-helix and b-sheet regions, respectively, within the AP2/ERF domain [62]. Asterisks represent amino acid residues that directly make contact with DNA [62]. Click here for file

Additional file 3 Outputs from the DESeq analysis using read distribution for contigs of each tissue-type libraries. Click here for file
