# Supplementary figures and images for: Identification of the Hevea brasiliensis AP2/ERF superfamily by RNA sequencing
Source: BMC Genomics. 2013 Jan 16;14:30. doi: 10.1186/1471-2164-14-30 (PMC3644242; doi:10.1186/1471-2164-14-30)

## Slide 1
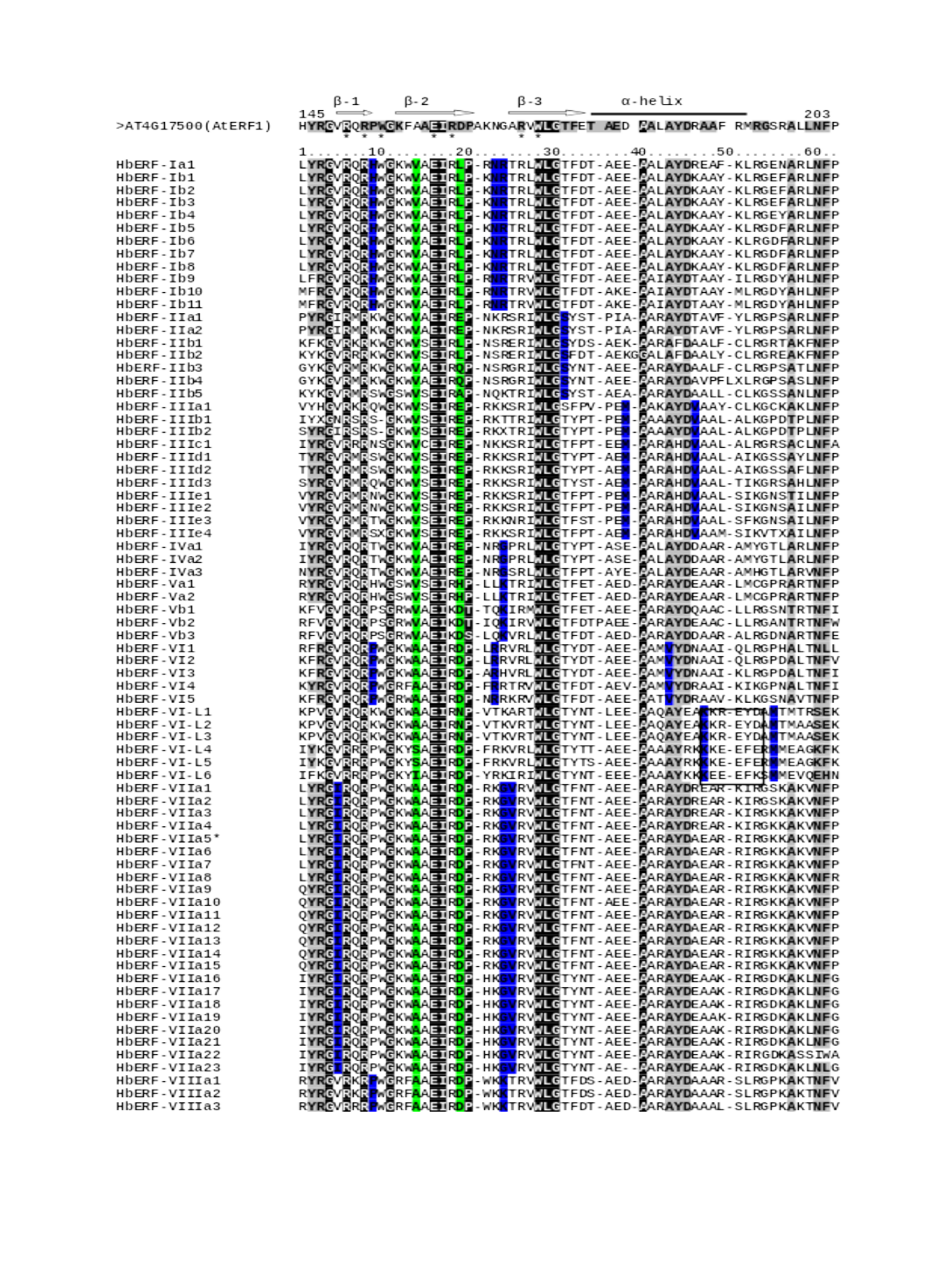

Supplement: Additional file 2: Figure S1 — Alignment of the AP2/ERF domains from H.brasiliensis 115 ERF family proteins. Black and light gray shading indicate identical and conserved amino acid residues, respectively. Dark gray shading indicates conserved amino acid residues in group VI-L. Green color indicates the V14, E19 residue conserved [39]; blue color indicates the residue conserved in each group individually;pink color indicates the supplementary residue in group IX The black bar and block arrows represent predicted a-helix and b-sheet regions, respectively, within the AP2/ERF domain [62]. Asterisks represent amino acid residues that directly make contact with DNA [62]. [file 1471-2164-14-30-S2.ppt]
